# Supplementary material for: The prevention of adverse pregnancy outcomes by periodontal treatment during pregnancy (PROBE) intervention study—A controlled intervention study: Protocol paper
Source: PLoS One. 2024 May 13;19(5):e0302010. doi: 10.1371/journal.pone.0302010 (PMC11090325; doi:10.1371/journal.pone.0302010)
Supplement: S1 Checklist — (DOC) [file pone.0302010.s001.doc]

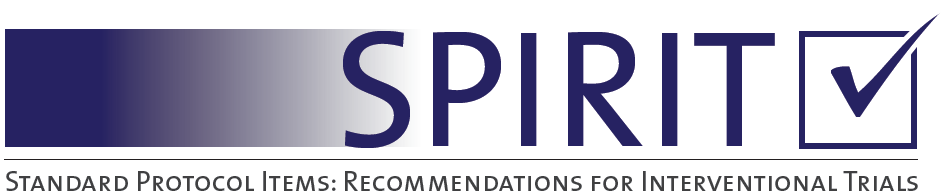


SPIRIT 2013 Checklist: Recommended items to address in a clinical trial protocol and related documents*

| Section/item | Item No | Description | Addressed on page number |
| --- | --- | --- | --- |
| **Administrative information** | | |  |
| Title | 1 | Descriptive title identifying the study design, population, interventions, and, if applicable, trial acronym | ____1_________ |
| Trial registration | 2a | Trial identifier and registry name. If not yet registered, name of intended registry | ____2_______ |
| Funding | 4 | Sources and types of financial, material, and other support | ____14______ |
| Roles and responsibilities | 5a | Names, affiliations, and roles of protocol contributors | ____1________ |
|  | 5b | Name and contact information for the trial sponsor | ____1_________ |
| Introduction |  |  |  |
| Background and rationale | 6a | Description of research question and justification for undertaking the trial, including summary of relevant studies (published and unpublished) examining benefits and harms for each intervention | ____4________ |
|  | 6b | Explanation for choice of comparators | ____5_________ |
| Objectives | 7 | Specific objectives or hypotheses | ____6_________ |
| Trial design | 8 | Description of trial design including type of trial (eg, parallel group, crossover, factorial, single group), allocation ratio, and framework (eg, superiority, equivalence, noninferiority, exploratory) | ___ 7_________ |
| Methods: Participants, interventions, and outcomes | | |  |
| Study setting | 9 | Description of study settings (eg, community clinic, academic hospital) and list of countries where data will be collected. Reference to where list of study sites can be obtained | __7, 8________ |
| Eligibility criteria | 10 | Inclusion and exclusion criteria for participants. If applicable, eligibility criteria for study centres and individuals who will perform the interventions (eg, surgeons, psychotherapists) | __5___________ |
| Interventions | 11 | Interventions for each group with sufficient detail to allow replication, including how and when they will be administered | __6___________ |
| Outcomes | 12 | Primary, secondary, and other outcomes, including the specific measurement variable (eg, systolic blood pressure), analysis metric (eg, change from baseline, final value, time to event), method of aggregation (eg, median, proportion), and time point for each outcome. Explanation of the clinical relevance of chosen efficacy and harm outcomes is strongly recommended | _7, 8___________ |
| Participant timeline | 13 | Time schedule of enrolment, interventions (including any run-ins and washouts), assessments, and visits for participants. A schematic diagram is highly recommended (see Figure) | __6___________ |
| Sample size | 14 | Estimated number of participants needed to achieve study objectives and how it was determined, including clinical and statistical assumptions supporting any sample size calculations | 6, 10______ |
| Recruitment | 15 | Strategies for achieving adequate participant enrolment to reach target sample size | ___8__________ |
| **Methods: Assignment of interventions (for controlled trials)** | | |  |
| Allocation: |  |  |  |
| Implementation | 16 | Who will generate the allocation sequence, who will enrol participants, and who will assign participants to interventions | ___5_____ |
| **Methods: Data collection, management, and analysis** | | |  |
| Data collection methods | 18 | Plans for assessment and collection of outcome, baseline, and other trial data, including any related processes to promote data quality (eg, duplicate measurements, training of assessors) and a description of study instruments (eg, questionnaires, laboratory tests) along with their reliability and validity, if known. Reference to where data collection forms can be found, if not in the protocol | _8-11_______ |
| Statistical methods | 19 | Statistical methods for analysing primary and secondary outcomes. Reference to where other details of the statistical analysis plan can be found, if not in the protocol | ___11-12_______ |
| Ethics and dissemination | | |  |
| Research ethics approval | 20 | Plans for seeking research ethics committee/institutional review board (REC/IRB) approval | _____2________ |
| Consent or assent | 21 | Who will obtain informed consent or assent from potential trial participants or authorised surrogates, and how (see Item 32) | ____5_________ |
| Confidentiality | 22 | How personal information about potential and enrolled participants will be collected, shared, and maintained in order to protect confidentiality before, during, and after the trial | ___13________ |
| Declaration of interests | 23 | Financial and other competing interests for principal investigators for the overall trial and each study site | ___14________ |
| Access to data | 24 | Statement of who will have access to the final trial dataset, and disclosure of contractual agreements that limit such access for investigators | ____15________ |
| Dissemination policy | 25 | Plans for investigators and sponsor to communicate trial results to participants, healthcare professionals, the public, and other relevant groups (eg, via publication, reporting in results databases, or other data sharing arrangements), including any publication restrictions | _____13_______ |

*It is strongly recommended that this checklist be read in conjunction with the SPIRIT 2013 Explanation & Elaboration for important clarification on the items. Amendments to the protocol should be tracked and dated. The SPIRIT checklist is copyrighted by the SPIRIT Group under the Creative Commons “[Attribution-NonCommercial-NoDerivs 3.0 Unported](http://www.creativecommons.org/licenses/by-nc-nd/3.0/)” license.
