# Supplementary material for: The prevention of adverse pregnancy outcomes by periodontal treatment during pregnancy (PROBE) intervention study—A controlled intervention study: Protocol paper
Source: PLoS One. 2024 May 13;19(5):e0302010. doi: 10.1371/journal.pone.0302010 (PMC11090325; doi:10.1371/journal.pone.0302010)
Supplement: S1 Appendix — (DOCX) [file pone.0302010.s002.docx]

**Appendix A** Data retrieved from questionnaires

| Data information | Timepoint | | | |
| --- | --- | --- | --- | --- |
|  | Questionnaire 1:  Nuchal fold scan (11-14 weeks of gestation) | Questionnaire 2:  35-36 weeks of gestation | Questionnaire 3:  2 months postpartum | Questionnaire 4:  6 months postpartum |
| Age | x |  |  |  |
| Pre-pregnancy weight | X |  |  |  |
| Number of visits at the dentist within the last 5 years | X |  |  |  |
| Last visit at the dentist | X | X |  | x |
| Reasons to not see a dentist regularly | X |  |  |  |
| Latest treatment at the dentist | X | X |  | x |
| Reason to last dental visit | X | X |  | x |
| Description of own teeth | X | x |  | x |
| Description of own gums | X | X |  | x |
| Complications with teeth or gums during pregnancy | X | X |  |  |
| Tooth brushing habits |  |  |  | x |
| Education | X |  |  |  |
| Employment | X |  |  |  |
| Working hours pr week | X |  |  |  |
| Civil status | X |  |  |  |
| Number of children | X |  |  |  |
| Previous history of preterm birth | X |  |  |  |
| Previous history of preeclampsia | X |  |  |  |
| Previous history of gestational diabetes | X |  |  |  |
| Ethnicity | X |  |  |  |
| Partner ethnicity | X |  |  |  |
| Emotional status and concerns within the last months | X | x |  |  |
| Concerns within the last 3 months | X | X |  |  |
| Intake of meat, fish, dietary products, fruit, vegetables, lentils, bread, sugar sweetened beverages, cake and candy | X | x |  |  |
| Intake of alcohol before pregnancy (amount) | X | x |  |  |
| Intake of alcohol during pregnancy (amount) | X | x |  |  |
| Smoking before pregnancy | X | x |  |  |
| Smoking during pregnancy (number of cigarettes) | X | x |  |  |
| Sleeping habits | X | x |  |  |
| Hours of sleep | X | x |  |  |
| Intake of medicine during the last 14 days | X | X |  |  |
| Assessment of health status in general | X | x |  |  |
| Any pain or discomfort within the last 14 days | X | x |  |  |
| Activity level | X | X |  |  |
| Planned pregnancy | X |  |  |  |
| Fertility treatment | X |  |  |  |
| Previous history of abortion | X |  |  |  |
| History of sick leave during pregnancy | X | x |  |  |
| Diagnose with gestational diabetes |  | x |  |  |
| Diagnose with preeclampsia |  | x |  |  |
| DOB |  |  |  | x |
| Weight of baby |  |  | x | x |
| Length of baby |  |  | x | x |
| Head circumference of baby |  |  | x | x |
| Breastfeeding |  |  | x | x |
| Duration of breastfeeding |  |  |  | x |
| Use of formula |  |  |  | x |
| Introduction to food (baby) |  |  |  | x |
| Biological role of partner to the baby |  |  | x |  |
| Partner weight |  |  | x |  |
| Partner height |  |  | x |  |
| Partner education |  |  | x |  |
| Partner employment |  |  | x |  |
| Mothers current weight |  |  | x | x |
| Opinions and satisfaction towards participating in the study |  |  |  | x |
