# Supplementary material for: The prevention of adverse pregnancy outcomes by periodontal treatment during pregnancy (PROBE) intervention study—A controlled intervention study: Protocol paper
Source: PLoS One. 2024 May 13;19(5):e0302010. doi: 10.1371/journal.pone.0302010 (PMC11090325; doi:10.1371/journal.pone.0302010)
Supplement: Attachment — Submitted filename: Response to Reviewers.docx [file pone.0302010.s003.docx]

**Response to Reviewers**

| **Journal Requirements:** | Responds |
| --- | --- |
| 1. Please ensure that your manuscript meets PLOS ONE's style requirements, including those for file naming. | Thank you for this comment. We have edited the manuscript so that it meets PLOS ONE’s style requirements. |
| 2. We note that the grant information you provided in the ‘Funding Information’ and ‘Financial Disclosure’ sections do not match. When you resubmit, please ensure that you provide the correct grant numbers for the awards you received for your study in the ‘Funding Information’ section. | We have now corrected the funding information. Please note that the received grant from Steno Diabetes Center Sjaelland have no grant number. |
| 3. Please provide a complete Data Availability Statement in the submission form, ensuring you include all necessary access information or a reason for why you are unable to make your data freely accessible. If your research concerns only data provided within your submission, please write "All data are in the manuscript and/or supporting information files" as your Data Availability Statement. | We have now provided a complete Data Availability Statement in the submission form. |
| 4. PLOS requires an ORCID iD for the corresponding author in Editorial Manager on papers submitted after December 6th, 2016. Please ensure that you have an ORCID iD and that it is validated in Editorial Manager. To do this, go to ‘Update my Information’ (in the upper left-hand corner of the main menu), and click on the Fetch/Validate link next to the ORCID field. This will take you to the ORCID site and allow you to create a new iD or authenticate a pre-existing iD in Editorial Manager. Please see the following video for instructions on linking an ORCID iD to your Editorial Manager account: <https://www.youtube.com/watch?v=_xcclfuvtxQ> | The corresponding author has an ORCID iD and it is now validated in Editorial Manager. |
| 5. Please include your full ethics statement in the ‘Methods’ section of your manuscript file. In your statement, please include the full name of the IRB or ethics committee who approved or waived your study, as well as whether or not you obtained informed written or verbal consent. If consent was waived for your study, please include this information in your statement as well. | The ethics statement including the full name of the ethics committee has now been included in the ‘Methods’ section, line 110. Obtained informed written consent has been added, please see line 119. Similar information has been updated in the submission form. |

Reviewer's Responses to Questions

**Comments to the Author**

1. Does the manuscript provide a valid rationale for the proposed study, with clearly identified and justified research questions?

The research question outlined is expected to address a valid academic problem or topic and contribute to the base of knowledge in the field.

Reviewer #1: Yes

Reviewer #2: Yes

Reviewer #3: Yes

2. Is the protocol technically sound and planned in a manner that will lead to a meaningful outcome and allow testing the stated hypotheses?

The manuscript should describe the methods in sufficient detail to prevent undisclosed flexibility in the experimental procedure or analysis pipeline, including sufficient outcome-neutral conditions (e.g. necessary controls, absence of floor or ceiling effects) to test the proposed hypotheses and a statistical power analysis where applicable. As there may be aspects of the methodology and analysis which can only be refined once the work is undertaken, authors should outline potential assumptions and explicitly describe what aspects of the proposed analyses, if any, are exploratory.

Reviewer #1: Yes

Reviewer #2: Yes

Reviewer #3: Partly

3. Is the methodology feasible and described in sufficient detail to allow the work to be replicable?

Descriptions of methods and materials in the protocol should be reported in sufficient detail for another researcher to reproduce all experiments and analyses. The protocol should describe the appropriate controls, sample size calculations, and replication needed to ensure that the data are robust and reproducible.

Reviewer #1: Yes

Reviewer #2: Yes

Reviewer #3: No

4. Have the authors described where all data underlying the findings will be made available when the study is complete?

The [PLOS Data policy](https://journals.plos.org/plosone/s/materials-and-software-sharing) requires authors to make all data underlying the findings described in their manuscript fully available without restriction, with rare exception, at the time of publication. The data should be provided as part of the manuscript or its supporting information, or deposited to a public repository. For example, in addition to summary statistics, the data points behind means, medians and variance measures should be available. If there are restrictions on publicly sharing data—e.g. participant privacy or use of data from a third party—those must be specified.

Reviewer #1: No

Reviewer #2: No

Reviewer #3: Yes

5. Is the manuscript presented in an intelligible fashion and written in standard English?

PLOS ONE does not copyedit accepted manuscripts, so the language in submitted articles must be clear, correct, and unambiguous. Any typographical or grammatical errors should be corrected at revision, so please note any specific errors here.

Reviewer #1: Yes

Reviewer #2: Yes

Reviewer #3: Yes

6. Review Comments to the Author

Please use the space provided to explain your answers to the questions above and, if applicable, provide comments about issues authors must address before this protocol can be accepted for publication. You may also include additional comments for the author, including concerns about research or publication ethics.

You may also provide optional suggestions and comments to authors that they might find helpful in planning their study.

| **Reviewer #1:** | Responds |
| --- | --- |
| Thank you for presenting your protocol. I have only one suggestion: Primary outcomes should include the rates of preterm birth (below 37 weeks and below 34 weeks), because this is a more clinically useful parameter, and should improve your work acceptance. | Thank you for reviewing the manuscript and for the relevant suggestion. We have now revised the manuscript, line 176-177. |
| **Reviewer #2:** |  |
| A two-arm controlled study is being proposed where the aim is to determine whether periodontal treatment during pregnancy can reduce systemic inflammation and lower risk of complications during pregnancy. | Thank you for taking the time to review the manuscript. |
| Minor revisions: |  |
| 1- Abstract: “The aim of this study is to determine whether periodontal treatment during pregnancy can reduce systemic inflammation and lower risk of complications during pregnancy and fetal growth.” The “and fetal growth” phrase in this sentence is confusing. | Thank you for this comment. We have made the aim in the abstract more clear, line 32. |
| 2- In the abstract, clarify if the study will be randomized or not. | We have added that the study is non-randomized, line 34. |
| 3- The standard statistical term for average is mean. | Thank you for pointing this out. We have changed average to mean, please see line 192 and 193. |
| 4- Line 226: Power calculation. Indicate the statistical testing method used to estimate the power in the scenarios. | Please see line 236. |
| 5- Line 240: List the confounders. | We have listed the potential confounders, line 250. |
| 6- Line 240: Describe the statistical methods that will be applied to analyze changes in hormonal and inflammatory markers. | We have added the statistical method that will be applied, please see line 251. |
| 7- Procedure for testing an interaction effect: If the interaction effect is significant, provide an interpretation of the results, but do not test main effects because the tests for main effects are uninteresting in light of significant interactions. If interaction effects are non-significant, drop the interaction effects from the model and test the main effects. Determining which results to present when testing interactions is often a multi-step process. | Thank you for this comment. We have inserted the text, please see line 254. |
| 8- Identify the software that will be used for statistical analysis. | The used statistical software is SPSS, (version 19, Chicago IBM) and mentioned on line 259. |
| **Reviewer #3:** |  |
| In the manuscript entitled: " The prevention of adverse pregnancy outcomes by periodontal treatment during pregnancy (PROBE) intervention study - A controlled intervention study: protocol paper" the authors aimed to assess whether periodontal treatment during pregnancy can reduce systemic inflammation and lower risk of complications during pregnancy and fetal growth. The authors concluded that Furthermore, the results will be communicated via social media, presented at academic meetings, and shared with participating communities as well as with national and international policymaking bodies. The REDCap platform, which will be used to collect demographic data, measures from the periodontal examinations, and data from the qualitative questionnaires, is a secure, web-based application designed to enable responses to be automatically uploaded into safe data storage to maximize data security. | Thank you for taking the time to review the manuscript. |
| Major comments: |  |
| In general, the idea and innovation of this study regards the analysis of oral and periodontal health in pregnancy is interesting and novel because the role these aspects in medicine are validated but further studies on this topic could be an innovative issue in this field could be open a creative matter of debate in literature by adding new information. Moreover, there are few reports in the literature that studied this interesting topic with this kind of study design. The study was well conducted by the authors; However, there are some concerns to revise that are described below. | Thank you for this comment. |
| The introduction section resumes the existing knowledge regarding the important factor linked with the impact mediators involved together oral health and with periodontitis. However, as the importance of the topic, the reviewer strongly recommends, before a further re-evaluation of the manuscript, to update the literature through read, discuss and must cites in the references with great attention all of those recent interesting articles, that helps the authors to better introduce and discuss the role of mediators (NT-PRO-BNP and TGF beta 1) in periodontitis and related recessions by adding as a references these article, before any further assessment of the manuscript: 1) DOI: doi: 10.1002/JPER.23-0063. PMID: 37433155; 2) DOI: 10.1177/1721727X1301100217 | Thank you for suggesting additional literature. We are however a bit confused about the suggested literature as the specific mediators (NT-PRO-BNP and TGF beta 1) are related to early risk of CVD and not pregnancy and birth related outcomes, which is the outcome measures in this protocol paper. We aim to be aligned with what we have uploaded on clinical trials.gov and as the suggested literature is outside the scope of the protocol of this study, we have not added the suggested literature. |
| The authors should be better specified, at the end of the introduction section, the rationale of the study and the aim of the study. In the central section, should better clarify inclusions and exclusions criteria of the selected sample. | We have now further highlighted the rationale and aim of the study, line 103. Inclusion and exclusion criteria have now been clarified, please see line 120-124. |
| Please better state the results obtained in the abstract. | Thank you for this comment. We have now added the planned outcome measures in the abstract, line 39 |
| The discussion section appears well organized with the relevant paper that support the conclusions, even if the authors should better discuss the relationship regarding the by periodontitis in and risk of oxidative stress evolution that could improve the quality of life in periodontitis patients which undergo pregnancy. The conclusion should reinforce in light of the discussions. | Thank you for suggesting this. However, we have no intention of measuring oxidative stress and thus finds it difficult, and outside the scope of the protocol, to include in the protocol paper. |
| In conclusion, I am sure that the authors are fine clinicians who achieve very nice results with their adopted protocol. However, this study, in my view does not in its current form satisfy a very high scientific requirement for publication in this journal and requests a revision before a further re-evaluation of the manuscript. | We have now revised the manuscript in view of your comments to the manuscript. |
| Minor Comments: |  |
| Abstract: -Better formulate the abstract section by better describing the aim of the study | The abstract has now been revised, see line 32-33. |
| Introduction: -Please refer to major comments | See comments to your comments above. |
| Discussion -Please add a specific sentence that clarifies the results obtained in the first part of the discussion | Thank you for this suggestion. As mentioned above we have now added outcomes measures in the abstract. As this is a protocol paper, we do not yet have results from the study. |
